# Modelling in vitro gametogenesis using induced pluripotent stem cells: a review

**DOI:** 10.1186/s13619-023-00176-5

**Published:** 2023-10-16

**Authors:** Maria Victoria Romualdez-Tan

**Affiliations:** 1 Present Address: Repro Optima Center for Reproductive Health, Inc., Ground Floor JRDC Bldg. Osmena Blvd. Capitol Site, Cebu City, 6000 Philippines; 2 Cebu Doctors University Hospital, Cebu City, Philippines

**Keywords:** In-vitro gametogenesis, Artificial gametes, Germ cell derivation, Primordial germ cell-like cells, Induced pluripotent stem cells

## Abstract

In vitro gametogenesis (IVG) has been a topic of great interest in recent years not only because it allows for further exploration of mechanisms of germ cell development, but also because of its prospect for innovative medical applications especially for the treatment of infertility. Elucidation of the mechanisms underlying gamete development in vivo has inspired scientists to attempt to recapitulate the entire process of gametogenesis in vitro. While earlier studies have established IVG methods largely using pluripotent stem cells of embryonic origin, the scarcity of sources for these cells and the ethical issues involved in their use are serious limitations to the progress of IVG research especially in humans. However, with the emergence of induced pluripotent stem cells (iPSCs) due to the revolutionary discovery of dedifferentiation and reprogramming factors, IVG research has progressed remarkably in the last decade. This paper extensively reviews developments in IVG using iPSCs. First, the paper presents key concepts from groundwork studies on IVG including earlier researches demonstrating that IVG methods using embryonic stem cells (ESCs) also apply when using iPSCs. Techniques for the derivation of iPSCs are briefly discussed, highlighting the importance of generating transgene-free iPSCs with a high capacity for germline transmission to improve efficacy when used for IVG. The main part of the paper discusses recent advances in IVG research using iPSCs in various stages of gametogenesis. In addition, current clinical applications of IVG are presented, and potential future applications are discussed. Although IVG is still faced with many challenges in terms of technical issues, as well as efficacy and safety, novel IVG methodologies are emerging, and IVG using iPSCs may usher in the next era of reproductive medicine sooner than expected. This raises both ethical and social concerns and calls for the scientific community to cautiously develop IVG technology to ensure it is not only efficacious but also safe and adheres to social and ethical norms.

## Background

In vivo gametogenesis is a complex process, and an extensive understanding of its molecular mechanisms is crucial for understanding reproductive health and associated diseases, such as infertility. Unfortunately, studies on germ cells raise several ethical issues (Aoi 2016). There is also the problem of scarcity of source materials for research, which has hindered the elucidation of mechanisms of germ cell development (Hong et al. 2021). These concerns have driven research in the direction of in vitro reconstitution of gametes, also known as in vitro gametogenesis (IVG), and the use of nonembryonic sources of cells and tissues for such research. The creation of properly functioning gametes in vitro not only allows further exploration of mechanisms of germ cell development, it also offers many possibilities in reproductive medicine, particularly for disease modelling and also for the potential of generating healthy offspring from individuals who cannot produce their own gametes in vivo (Saitou and Hayashi 2021).

Earlier studies on IVG involved the use of pluripotent stem cells (PSCs) of embryonic origin. However, this approach does not obviate the problem of scarcity and the ethical issue of using embryonic cells. A novel approach that overcomes these concerns is the use of induced pluripotent stem cells (iPSCs) rather than PSCs of embryonic origin (Nishikawa et al. 2008). The first iPSCs were derived from skin fibroblasts, but because iPSCs can be derived from any differentiated somatic cell, other abundant sources such as peripheral blood cells, keratinocytes, and even cells in the urine can be reprogrammed into iPSCs (Liu et al. 2020; Singh et al. 2015).

Even while using iPSCs, many of the studies on IVG use mouse models. The molecular mechanisms of gamete development, however, differ among species (Stirparo et al. 2018). For instance, Sox17, a key regulator of human primordial germ cells (hPGCs), is only transiently expressed in mouse primordial germ cells (mPGCs) (Irie et al. 2015). Another difference is in the Sox2 expression which is downregulated in hPGCs but regained in mPGCs (Sasaki et al. 2015). The activity of Blimp1 also varies between mPGCs and hPGCs where it suppresses the somatic mesodermal program in the former while it inhibits the program for neuron development in the latter (Sasaki et al. 2015). Despite these differences, murine models are extremely useful given their genetic malleability and the ability to observe the development of engineered cells in vivo with markers. Moreover, several studies on IVG have shown that the processes involved in the creation of in vitro derived human gametes are in many ways similar to the processes involved in the generation of in vitro induced mouse gametes using iPSCs (Luo and Yu 2022).

This paper extensively reviews IVG using iPSCs in mouse and human models. After laying down key concepts from foundation studies on IVG, the paper briefly discusses techniques for deriving iPSCs and recent strategies employed to improve its efficacy when used for IVG. The main portion of this paper walks the readers through IVG research using iPSCs and the methodologies used to generate male and female gametes in every stage of gamete development. In doing so, this review provides a roadmap to understanding the present status of IVG research in mice and humans, and a perspective on its current and potential clinical applications, the challenges it is faced with, and the direction towards which IVG should be taken.

## In vitro gametogenesis: key concepts and foundation studies

Several studies have aimed to generate gametes in vitro from PSCs in both mice and humans (Daley 2007; Saitou and Yamaji 2010). In mice, two types of in vivo pluripotent cells give rise to two distinct types of pluripotent stem cells in vitro. Specifically, the ICM of preimplantation blastocysts at E3.5-4.5 gives rise to embryonic stem cells (ESCs), which are in the *naïve* or ground state and can contribute to all lineages, including chimaeras and germ cells, while the epiblasts of post-implantation blastocysts at E5.5-6.5 give rise to epiblast stem cells (EpiSCs), which are in the primed state with a biased differentiation potential and are thus recalcitrant to germ cell differentiation and chimaera formation (Brons et al. 2007; Evans and Kaufman 1981; Hayashi and Surani 2009; Tesar et al. 2007). ESCs and EpiSCs also have distinct genetic and epigenetic profiles, cytokine dependencies, and morphologies (Hayashi et al. 2011). *Naïve* mESCs express naïve pluripotency genes, such as *Klf2/4/5*, *Tfap2C*, *Esrrb*, *Tbx3*, and *Zfp42*, and the core pluripotency genes (*Oct4*, *Nanog*, and *Sox2*). Primed mEpiSCs, on the other hand, express genes encoding primed transcription factors, such as *Oct6*, *Otx2*, *Sox2*, *Bex1* and *Tead2*, as well as the core pluripotency genes (Hackett and Surani 2014).

In early attempts at IVG, ESCs were used to produce primordial germ cells (PGCs) using embryoid bodies that spontaneously differentiated under undefined conditions (Geijsen et al. 2004; Hübner et al. 2003; Toyooka et al. 2003). However, the efficiency of PGC formation was low, even when culture media had been added BMP4, a molecule that signals proximal epiblast cells to acquire potential competence to become PGCs (Toyooka et al. 2003). Subsequent attempts used EpiSCs to produce PGC-like cells (PGCLCs) in vitro because EpiSCs were thought to be the in vitro equivalent of the epiblast. The results of these studies, however, showed that less than 1.5% of *Blimp1*-positive cells also expressed the PGC-specific marker *Stella* (Hayashi and Surani 2009). This study showed that EpiSCs are insufficient to generate PGCLCs. Moreover, in mice, neither of the pluripotency states has PGC competence because competence is conferred only temporarily during the transition from the *naïve* to the primed state (Hayashi and Surani 2009; Ying et al. 2003). Human ESCs (hESCs) are more similar to mouse EpiSCs and are in a primed state of pluripotency, which exemplifies the difficulty of achieving cells in a *naïve* state of pluripotency from nonrodent species (Nichols et al. 2009).

Because earlier experiments that attempted to create PGCs through embryoid body (EB) formation using random differentiation strategies resulted in very inefficient and inconsistent PGC specification (only approximately 0.5–3.6% per EB), a direct induction method was deemed necessary for PGC specification in vitro (Ohinata et al. 2009). In 2009, Ohinata et al. became the first to establish such conditions for mouse epiblast cells. Epiblasts of the pregastrula were cultured in BMP4/8b, stem cell factor (SCF), epidermal growth factor (EGF), and leukaemia inhibitory factor (LIF) for the induction of *Blimp1* and *Prdm14* expression. The resultant epiblast-derived *Blimp1-mVenus-* and *Stella-ECFP*-positive PGC-like cells (epiPGCs) had genetic and epigenetic signatures consistent with those of in vivo PGCs. Upon transplantation into neonatal testes, these epiPGCs developed into mature spermatozoa capable of fertilizing oocytes and producing healthy offspring (Ohinata et al. 2009). This study laid the groundwork for the signal interactions needed for PGC specification from pluripotent stem cells and showed that the direct in vivo precursor of PGCs is the epiblast. This prompted other researchers to use PSCs to produce PGC-like cells (PGCLCs) in vitro, including EpiSCs, since they retain attributes of the original epiblast and can potentially give rise to germ cell-like cells in vitro (Hayashi and Surani 2009; Tesar et al. 2007).

In an experiment using EpiSCs to generate in vitro gametes conducted in 2009, Hayashi and Surani showed that only a small population of EpiSCs express *Blimp1* under self-renewing conditions, and only a minority of these cells are *Stella*-positive. Moreover, only approximately 1.5% of these *Blimp1* expressing, *Stella-*positive cells emerge from EpiSCs even when cultured in BMP4 (Hayashi and Surani 2009). This low frequency of PGC induction from EpiSCs demonstrates that EpiSCs acquire properties that prevent the efficient derivation of PGCs in culture. This finding is in agreement with Ohinata’s observation that the competence of the epiblast to become PGCs is markedly reduced after approximately E6.25 (Ohinata et al. 2009).

It became evident that to increase the efficiency of PGC specification for PSCs, they should be maintained in a population that is homogeneously germline competent (Saitou and Hayashi 2021). Ying et al. (2008) advocated culturing PSCs in MAPK and GSK (referred to as 2i) and LIF, or 2i + LIF, to maintain them as germline competent *naïve* PSCs, a condition called the ground state. Based on the gene expression profile, the PSC ground state is equivalent to that of the E4.5 preimplantation epiblast (Marks et al. 2012).

In 2011, Hayashi et al. were the first to describe the stepwise induction of mPSCs to PGCLCs. They obtained *Blimp1-mVenus-* and *Stella-ECFP* (BVSC)-positive ESCs from E3.5b mouse blastocysts and maintained these cells in the ground state using 2i + LIF feeder-free culture. These ESCs were then stimulated with ActA, bFGF, and 1% KSR for 2 days to produce what they called epiblast-like cells (EpiLCs). Based on an analysis of the expression of key genes in day 2 EpiLCs, they demonstrated that these cells had properties that corresponded to those of E5.75 pre-gastrulating epiblasts, thus suggesting that they were germline competent precursors capable of differentiation into PGCLCs (Hayashi et al. 2011). These 2d EpiLCs are therefore intermediate precursors for PGC formation and likewise capable of being induced into other cell lineages that arise from pre-gastrulating epiblasts. Further on in the experiment, Hayashi et al. demonstrated that culturing these EpiLCs in the same conditions described by Ohinata et al. in 2009, i.e., using BMP4, BMP8b, EGF, SCF and LIF, induced these EpiLCs to become PGCLCs (Hayashi et al. 2011; Ohinata et al. 2009). After transplantation into germ cell-deficient *W/W*^*v*^ mice, these PGCLCs demonstrated proper spermatogenesis in the host testes with the resultant sperm capable of fertilizing an oocyte after ICSI, eventually producing healthy offspring. Although teratomas were detected in some of the host testes, sorting by SSEA1 and Integrin-β3 was shown to produce a purer PGCLC population with no significant contamination of teratogenic cells (Hayashi et al. 2011).

In the same study, Hayashi et al. (2011) also used induced pluripotent stem cells (iPSCs) to explore whether the same germ cell specification pathway would induce iPSCs to become PGCLCs. Further studies using three iPSC lines from mice revealed that although all lines initially bore *Nanog*-*Egfp* (NG) transgenes and expressed NG in the ground state, the three lines exhibited different SSEA1 and Integrinβ-3 FACS sorting patterns at day 6. Only one iPSC line (20D17) was very similar to the ESC lines. Likewise, only this 20D17 line exhibited proper spermatogenesis after being transplanted into *W/W*^*v*^ mouse testes. These findings show that despite iPSCs exhibiting different induction patterns depending on the iPSC line, they can nevertheless be induced into PGCLCs that function properly (Hayashi et al. 2011). Elucidation of the properties of the 20D17 line by Okita et al. in 2008 revealed that this iPSC cell line was derived by stable retroviral transduction of *c-Myc,* while the other two lines were without, or only transiently with, *c-Myc* (Nakagawa et al. 2008; Okita et al. 2007). Therefore, the efficiency of germline induction of iPSCs depends largely on the original properties of the iPSC lines, particularly regarding the incorporation of certain crucial transgenes (Hayashi et al. 2011).

The capacity to generate properly functioning PGCLCs from iPSCs was a breakthrough in the study of IVG. Many of the studies since those by Hayashi’s group in 2011 have used iPSCs, and approaches that are effective for ESCs can universally be applied to iPSCs.

## Induced pluripotent stem cells for in vitro gametogenesis

The emergence of induced pluripotent stem cells (iPSCs) has presented a promising alternative to cells of embryonic origin, making it possible to elucidate aspects of germ cell biology that have been unexplored due to material limitations and ethical concerns (Hayashi et al. 2011). As iPSCs are derived from differentiated somatic cells found in samples such as blood, skin and urine, the sources of iPSCs are abundant and easy to obtain via non-invasive methods (Liu et al. 2020).

**Fig. 1:**
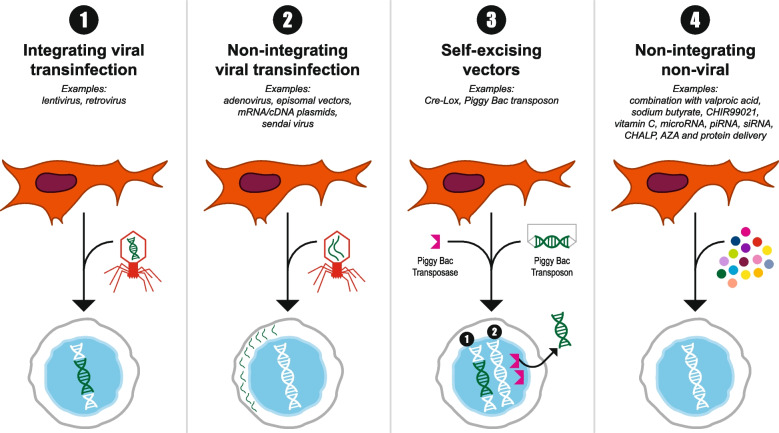
Key methods for introducing reprogramming factors. Whereas integrating viral systems (1) were the first to be used, it incorporated viral genetic material that caused teratoma. Newer methods (2–4) avoid this and significantly improves the safety and efficacy of iPSCs especially for clinical applications. (Modified from Liu et al. 2020)

The generation of iPSCs from mouse fibroblasts was first described by Yamanaka and colleagues in 2006 (Takahashi and Yamanaka 2006). A year later, the same group reported the generation of human iPSCs (hiPSCs) from fibroblasts (Takahashi et al. 2007). Traditionally, iPSCs were created using viral vectors, specifically retroviruses. However, this created iPSCs with inadequate expression of native pluripotency genes (Sridharan et al. 2009). Lentiviral vectors have the best reprogramming efficiency, between 0.1% and 1% (Stadtfield et al. 2010), but nonviral integrative systems for nuclear reprogramming have also been used (Lee et al. 2017; Okita et al. 2007). This strategy, which ensures safety for therapeutics, involves the use of two plasmids (Okita et al. 2007), one encoding *c-Myc* and the other encoding the four crucial reprogramming factors *Oct4, Sox2, c-Myc,* and *Klf4* (together, these four factors are often referred to as OSKM). Although OSKM have been the four most popular reprogramming factors*,* iPSCs have also been generated using the expression of *Oct-4, Sox2, Nanog,* and *Lin28* (Yu et al. 2014). These systems, however, showed a risk of integration and had low reprogramming efficiency (Okita et al. 2007). Nonintegrative nonviral reprogramming systems have also been employed using self-replicating vectors and cytoplasmic RNA. This has, however, a lower reprogramming efficiency than the system using lentiviral vectors (Lee et al. 2017). More recently, successful reprogramming of somatic cells using microRNAs has been investigated and showed improved efficiency (Fig. 1). In this approach, *c-Myc* is substituted with miR-295, miR-294, and miR-291-3p to create uniform populations of hiPSCs (Lee et al. 2017). This indicates that a pluripotent ground state can be achieved through the activation of different transcription factors and/or miRNAs (Yu et al. 2014).

To study germ cell derivation using iPSCs, it is crucial that the iPSCs used have highly efficient germline transmission (Okita et al. 2007). Unfortunately, the newer and safer nonviral methods of introducing reprogramming factors into somatic cells to dedifferentiate them into transgene-free iPSCs could reduce their capacity to generate a functional germ line (Wu et al. 2014). In 2014, Capecchi’s group reported the use of optimally congregated reprogramming factors and positive/negative selection factors within single plasmids functioning as nonintegrating but stably transmissible episomes to produce germ-line competent iPSCs. To avoid the use of multiple episomes, they added the genes *LIN28, neo, HSVtk, NANOG, NR5A2* and the microRNA 302/367 gene cluster to the classic OSKM reprogramming factors to produce the pMaster12 episome vector (Sui et al. 2014). The iPSCs generated by the pMaster12 episome are transgene-free and, when cultured in 2i medium, resemble high-quality ESCs in their capacity to generate germ-line chimaeras (Wu et al. 2014).

The generation of transgene-free iPSCs with high capacity for germline transmission is crucial for IVG studies, as it could preclude the need to utilize embryonic-derived stem cells in those studies, thus overcoming the difficulties associated with their use.

## In vitro gametogenesis using iPSCs

### PGCLC derivation

As previously mentioned, in 2011, Hayashi et al. first explored the derivation of PGCLCs using murine iPSC lines (Hayashi et al. 2011). Soon after, Irie et al. recommended culture conditions that can be used to efficiently derive hPGCLCs from hiPSCs (Irie et al. 2015). To maintain hiPSCs in a near-ground state of pluripotency, they preconditioned the cells in 4 kinds of inhibitors (4i), including a MEK inhibitor (PD0325901), a JNK inhibitor (SP600125), a p38 MAPK inhibitor (SB203580) and a GSK3 inhibitor (CHIR99021). Then, medium containing bFGF/TGFβ was added to the culture for 2 days, and finally, the hiPSCs were placed in a suspension culture containing BMP2 or 4, SCF, EGF and LIF. This approach induced the hiPSCs into hPGCLCs that were similar to hPGCs in terms of epigenetic patterns and genetic expression. Further, the results suggested that the TF for endoderm specification, *SOX17*, may be crucial for hPGCLC specification, as very early expression (on day 1 of suspension culture) together with *BLIMP1* was noted. In association with *SOX17*, *BLIMP1* not only suppresses somatic differentiation by itself but also promotes germ cell specification (Irie et al. 2015).

The second group, Sasaki et al. (2015), argued that because hiPSCs preconditioned in 4i medium did not consistently exhibit *naïve* pluripotency markers, these hiPSCs were not truly in a ground state of pluripotency but instead represented a different cell type, incipient mesoderm-like cells (iMeLCs), as an intermediary step from hiPSCs to hPGLCs. The hiPSCs were first activated using a GSK3 inhibitor (CHIR99021) and activin A to induce iMeLCs that expressed pluripotency genes as well as genes for mesoderm development (EOMES, T/brachyury, MIXL1 and SP5). These cells were then placed in suspension culture using BMP4, EGF, SCF and LIF to convert the iMeLCs into BVSC-positive hPGCLCs that expressed Blimp1, Oct4, Tfap2c, Sox17 and Nanog, which are all early germ cell markers in humans. Gene Ontology analysis after RNA-seq showed that Sox17 and Blimp1 are crucial regulators of hPGCLC specification (Sasaki et al. 2015).

The addition of either activin A or vitamin C to the medium also improves the induction of hPGCLCs from hESCs/hiPSCs (Li et al. 2019b; Mishra et al. 2021). Vitamin C causes epigenetic changes by increasing the expression of TET (ten-eleven translocation), thereby enhancing germ cell differentiation (Li et al. 2019b). Activin A, on the other hand, is an established inducer of oogenesis in the foetus and after birth and is thus crucial for germ cell development (Wang et al. 2019).

### Derivation of spermatogonia and oogonia

#### Differentiation of spermatogonia

Earlier studies attempted to derive spermatogonia from PGCLCs by injecting them into neonatal testes. However, the natural niche of PGCs is the epiblast, hindgut and gonadal ridges of the prenatal fetus. Thus, the postnatal testis is not a suitable site for transplantation of PGCLCs for the purpose of generating sperm. Instead, the earliest type of germ cell in the postnatal testes is the spermatogonial stem cell (SSC). These SSCs can self-renew and produce haploid gametes and spermatocytes through association with Sertoli cells (DeRooij and Russel 2009). The in vitro culture of SSCs is possible at present, and the genes expressed by undifferentiated SSCs in culture are *Pax7*, *Vasa*, *BclB6*, *Etv5*, and *Gfra1* (Ahn et al. 2020). In 2006, Nayernia’s group was the first to attempt deriving SSCs in vitro; however, the resultant spermatids were not completely characterized, and it is unclear whether these cells were indeed spermatids (Nayernia et al. 2006). The Ishikura group was able to induce SSCs from iPSCs/ESCs using reconstituted testes comprising aggregates of PGCLCs and somatic cells from E12.5 foetal testes (Ishikura et al. 2016). After 21 days of culture, PLZF+ SSC-like cells were observed within the aggregates that had formed structures appearing like seminiferous tubules (Ishikura et al. 2016). Subsequent transplantation of these SSC-like cells into *W/W*^*v*^ adult mouse testes produced spermatids and spermatozoa capable of fertilizing oocytes through intracytoplasmic sperm injection (ICSI) that then produced embryos in a gestational mother (Ishikura et al. 2016; Saitou and Hayashi 2021).

In humans, Hwang et al. (2020) obtained pro-spermatogonia-like cells through prolonged air–liquid interface (ALI) culture of germ cells derived from hiPSCs aggregated with testicular somatic cells from mice. The resultant hPGCLCs from these xenogeneic aggregates developed into M (multiplying)-prospermatogonia on the 77^th^ day and T1 (primary transitional)-prospermatogonia on the 120^th^ day in the ALI system. Single-cell RNA-seq analysis of gene expression patterns showed that these in vitro*-*derived prospermatogonial cells were equivalent to their corresponding in vivo counterparts. However, the functionality of these cells was not assessed (Hwang et al. 2020).

#### Differentiation of oogonia

Human oogonia were recently generated in vitro by Yamashiro et al. (2018) using *BLIMP1*-*tdfTomato*+ and TFAP2C-EGFP+ hiPSCs through Sasaki’s method, where hPGCLCs were produced through intermediary iMeLCs (Sasaki et al. 2015; Yamashiro et al. 2018). The resultant FACS-sorted hPGCLCs were then aggregated with ovarian somatic cells from mouse embryos producing xenogeneic ovary structures. The structures became cyst-like after a week in culture. The *TFAP2C*-*EGFP*-positive hiPSCs differentiated into oogonia-like cells expressing *DDX4* and *DAZL* on the 77^th^ day in culture, resembling mouse granulosa cells. On the other hand, on the 120^th^ day of culture, the *BLIMP1*-tdTomato + /TFAP2c-EGFP+ hiPSCs developed into cells that corresponded with foetal germ cells expressing the meiotic initiating gene *STRA8* (EGFP-fused) but not the genes for meiotic recombination. The transcriptomic profiles of these oogonia-like cells from hiPSCs were akin to those of W7 oogonia and W9 gonocytes of embryos in humans (Tang et al. 2015; Yamashiro et al. 2018).

### Spermatogenesis

T﻿o achieve in vitro spermatogenesis, Zhou et al. proposed an induction procedure using the aggregate method (Zhou et al. 2016) to generate mPGCLCs based on an adaptation of Hayashi’s technique. However, instead of transplanting these cells, Zhou et al. (2016) replicated the in vivo conditions by aggregating the mPGCLCs with cells from the testes of recently born mice. These aggregates were cultured for 6 days in meiotic-inductive medium containing activin A, BMP2/4/7 and retinoic acid, which induced meiosis in the mPGCLCs, as evidenced by chromosome synapse formation. The addition of FSH, BPE (bovine pituitary extract), and testosterone induced the production of haploid SLCs (spermatid-like cells) containing a distinct acrosome and imprinting patterns in the *SNRPN* (small nuclear ribonucleoprotein polypeptide N) and H19 loci (Zhou et al. 2016).

Ishikura et al. (2016) described an efficient method of deriving SLCs from d4 mPGCLCs derived from mESCs (technically also applicable to miPSCs) aggregated with E12.5 testicular somatic cells (Ishikura et al. 2016). These cells continue as germline stem cell-like cells (GSCLCs), which possess self-renewal characteristics and can develop into mature gametes. GSCs can generate mature functional spermatozoa when transplanted into *W/W*^*v*^ mice (Kanatsu-Shinohara et al. 2005). Initial studies have shown that GSCLCs have sluggish and inefficient differentiation into SLCs (Ishikura et al. 2016). To improve the differentiation potential of these cells, Ishikura et al. (2021) used a *Ddx4*-controlled red fluorescent protein (RFP) + BVSC system. They showed that day 4 mPGCLCs in 5-day culture with FR10Cs5 (10 µM forskolin, 10 µM rolipram and 5 µM cyclosporin A) subsequently combined with E12.5 testicular somatic cells to form rTestes (reconstituted testes), which had the best results in producing SLCs according to the ratio and number of VR-positive cells in the ALI system for at least seven days. Once these SLCs are transplanted into testes and the testicular transplants are cultured in vitro, they differentiate into mature functional sperm (Ishikura et al. 2021).

At present, in vitro spermatogenesis can generate only up to the haploid spermatid stage; hence, in vitro spermiogenesis is the current bottleneck in achieving complete male IVG (Saitou and Hayashi 2021).

### Oogenesis

In 2016, Hayashi et al. were able to generate metaphase II oocytes from mouse ESCs/iPSCs in a completely in vitro environment (Hikabe et al. 2016). Shortly after their ground-breaking success in producing mPGCLCs from mouse iPSCs/ESCs through intermediate EpiLCs, they were able to differentiate these mPGCLCs further by aggregating them with E12.5 ovarian somatic cells (rOvaries) for subsequent transplantation into the bursa of immunodeficient mouse ovaries. In this process, the mPGCLCs transformed into GV (germinal vesicle)-stage oocytes, which then underwent in vitro maturation (IVM) and in vitro fertilization (IVF), resulting in fertile offspring with an efficiency of approximately 3.7% (Hayashi et al. 2012). To reconstitute oogenesis entirely in vitro, Hayashi et al. (2012) subdivided the steps between mPGCLCs to metaphase II oocytes into three phases: IVDi (in vitro differentiation), IVGr (in vitro growth), and IVM (in vitro maturation). Instead of transplanting the rOvaries, they extended their culture to approximately 5 weeks, which resulted in the formation of cumulus–oocyte complexes containing SC (*Stella*-controlled ECFP)-positive primary oocytes and *Foxl2*^+^ granulosa cells similar to those in primary follicles (Hayashi et al. 2017; Hikabe et al. 2016). For the IVG phase, these follicles were harvested from the rOvaries and cultured for 11 days, during which the primary oocytes differentiated into GV oocytes. These oocytes were then cultured in IVM medium. Approximately 28.9% of the oocytes extruded the first polar body to enter meiosis II and produce metaphase II oocytes, which produced healthy offspring after IVF. Although the live birth rate from these in vitro-derived oocytes is low, at only 3.5%, and meiotic and epigenetic flaws are present, this study is valuable for its capacity to reconstruct the entire process of oogenesis in vitro*,* allowing for the study of interactions between gonadal somatic cells and gametes (Miyauchi et al. 2017; Ohta et al. 2017). Another drawback of this study is the need to use embryonic somatic cells. The novel technique of producing fetal ovarian somatic cell-like cells (FOSLCs) from mESCs/miPSCs is promising and may obviate the need for embryonic somatic cells (Yoshino et al. 2021). Transcriptomic analysis of these FOSLCs showed that they express Nr5A1, the gene marker of gonadal somatic cells, and their cellular composition and transcription patterns correspond to E12.5 somatic cells of the ovary (Stévant et al. 2019). When aggregated with mPGCLCs, these FOSLCs formed reconstituted ovarioids (rOvarioids), which produced functional oocytes. This shows that FOSLCs are capable of supporting the differentiation of germ cell progenitors into mature and potentially functional oocytes, producing a model where later stages of oocyte development can be studied for future clinical applications (Yang and Ng 2021).

Another breakthrough in oogenesis research is the capacity to generate oocyte-like cells from direct induction using forced expression of transcription factors without the need for prior specification into PGCLCs (Hamazaki et al. 2021). Forced expression of TFs for the primordial to primary follicle transition (PPT), such as Figla, Dynll1, NOBOX, Tbpl2, Sub1, Stat3, Sohlh1, and Lhx8, produced follicle-like structures from aggregates of miPSCs/mESCs and E12.5 somatic cells from female fetal gonads. The oocyte-like cells formed in these follicle structures develop into metaphase II oocytes that were shown to produce early 8-cell embryos (Hamazaki et al. 2021).

In humans, forced expression of *DAZL* and *BOULE* led to the derivation of follicle-like cells (FLCs) from hiPSCs/hESCs (Jung et al. 2017; Kee et al. 2009). First, BMP4 and BMP8 were used to induce germ cell competence in hiPSCs/hESCs, followed by the induction of meiosis through overexpression of *DAZL* and *BOULE* using lentiviruses and the addition of BMP15 and GDF9. The resultant FLCs had oocyte-like cells covered by multiple layers of cells on the 9^th^ day after induction. These oocyte-like cells were akin to primordial oocytes based on transcriptome patterns and expressed *NOBOX*, *VASA*, *ZP2* and *AMH*. When these cells were transplanted into the kidney capsule, they formed primordial follicle-like structures, demonstrating that FLCs that develop from hiPSCs/hESCs are functional ovarian follicles. This in vitro FLC-derivation model can be useful in elucidating the early processes of human folliculogenesis and germline development (Jung et al. 2017).

**Fig. 2:**
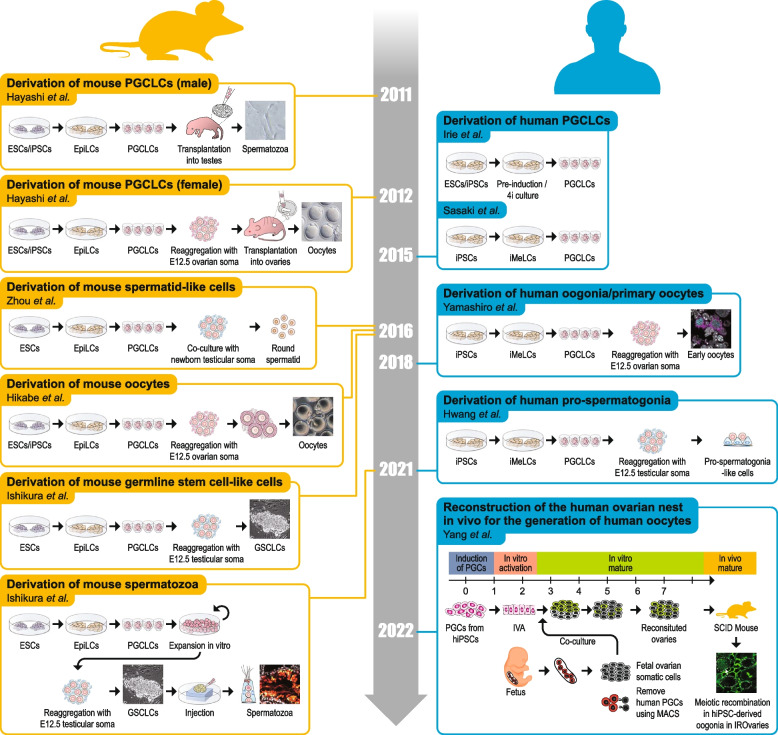
Roadmap of IVG research using ESC/iPSC in mice and humans. (Modified from Saitou and Hayashi 2021; Yang et al. 2022)

In a very recent study, Yang et al. were able to induce meiosis resumption in hPGCLCs from hiPSCs using in vitro activation and reconstruction ovarian nests called IrOvaries (isogeneic reconstituted ovaries) (Yang et al. 2022). These IrOvaries were formed by aggregating hPGCLCs and foetal ovarian somatic cells from 7- to 8-week-old aborted fetuses. In vitro activation (IVA) was performed by stimulation of the Wnt pathway. Their results showed that Wnt plays a crucial role in meiotic resumption and that regulation of *GSK-3* expression is important for regulating the timing of meiotic resumption. After IVA, the reconstituted ovaries were transplanted into SCID mice for folliculogenesis. This study provides a robust approach to differentiate hiPSCs into haploid oocytes in vitro and is among the more recent works done on IVG (Fig. 2).

## Emerging techniques

A promising development in the field of IVG is the creation of synthetic embryos using ESCs/iPSCs from which germ cells can then be derived in vitro (Kotsiliti 2022). Synthetic embryos (embryoids) are different from embryonic bodies, which are merely disorganized three-dimensional clusters of cells. Embryoids, on the other hand, have the correct topology and polarity of various cell types according to the stage of the embryo as defined by the extracellular matrix in the surrounding media (Stirparo et al. 2018).

### Blastoids

Rivron et al. in 2018 produced in vitro structures termed blastoids that resembled E3.5 blastocysts in appearance and transcription pattern (Rivron et al. 2018). To produce these blastoids, mESCs (technically also applicable to smiPSCs) were aggregated with mTSCs (trophoblast stem cells from mice) and sequentially seeded under three-dimensional suspension culture.

Since then, different types of blastoids have been produced in vitro using different kinds of stem cells and different growth factors and inhibitors (Stirparo et al. 2018). Blastoids from EPS (extended pluripotent stem cells, also derived from ESCs/iPSCs) include the Belmonte group’s EPS blastoid (Li et al. 2019a), Zernicka-Goetz group’s ZG blastoid or ETS-embryos (Sozen et al. 2019), iBLCs or blastocyst-like cysts (Kime et al. 2019), iBlastoids (Liu et al. 2021), the Fan group’s EPS blastoids (Fan et al. 2021), and human blastoids (Yanagida et al. 2021), to name a few. Although gametes have not yet been derived from these blastoids, PGC specification induced by BMP-SMAD signalling, such as that in the ETS-embryos of the Zernicka-Goetz group, has been demonstrated (Amadei et al. 2021).

### Gastruloids

By incorporation of extraembryonic endoderm cells into ESCs/iPSCs and TSCs, ETS-embryos develop into gastruloid-like E7.0 embryos at mid-gastrulation (Sozen et al. 2018). Mesodermal specialization and asymmetric patterning are more efficient in ETX-gastruloids than in ETS-embryos, and PGC specification is demonstrated at the posterior portion of the junction between TSC and ESC compartments (Sozen et al. 2018). As with blastoids, it is uncertain whether germline development can proceed beyond PGC specification in these gastruloids. However, these studies have established that the creation of synthetic embryos is another way of obtaining PGCs from iPSCs/ESCs in vitro (Saitou and Hayashi 2021).

## Clinical applications

One of the promising applications of IVG using iPSCs is for disease modelling to characterize fundamental pathogenetic mechanisms of diseases, thereby paving the way for a range of new approaches to treatment (Hayashi et al. 2012). To date, in vitro gametes from iPSCs of patients with premature ovarian insufficiency (POI) and nonobstructive azoospermia (NOA) have been studied to understand their pathogenesis on a cellular basis (Leng et al. 2015).

Leng et al. obtained iPSCs from POI patients with Xq deletions to study their differentiation potential (Leng et al. 2015). *VASA-GFP* reporter genes were transfected into these POI-hiPSCs to monitor germ cell development, and the cells were treated with WNT3a or BMP4 to induce PGC differentiation. *VASA*-*GFP*-positive cells were found to express the early germ cell markers *DAZL*, *BLIMP1*, and *DPPA3* but not the meiotic marker *SCP3*. This indicates that POI-hiPSCs could differentiate into premeiotic PGCs (Leng et al. 2015). Moreover, five genes associated with germ cell development in the Xq region were found to be significantly downregulated in these *VASA*-*GFP*-positive cells, and these genes were concluded to potentially be the main aetiology of the disease (Leng et al. 2015; Tilgner et al. 2010).

In 2018, Zhao et al. used their protocol for deriving *PLZF*-positive spermatogonium-like cells from hiPSCs in NOA patients (Zhao et al. 2018). They found that hiPSCs from patients with NOA due to Sertoli-only syndrome produced PLZF-positive spermatogonium-like cells less efficiently, while those with NOA due to AZFc microdeletions presenting with only mild symptoms had normal production (Zhao et al. 2018). These findings suggest that the capacity to produce spermatogonia-like cells from NOA-hiPSCs could potentially be used to diagnose the type and aetiology of male infertility (Saitou and Hayashi 2021). In another study, Fang et al. derived hPGCLCs through iMeLCs from NOA-hiPSCs, and the resulting cell population showed low expression of PGC markers and a high proportion of apoptotic cells compared to the population from hiPSCs of normal individuals. They concluded that the poor development of NOA-hiPSCs into hPGCLCs is caused by apoptosis during PGC specification (Fang et al. 2020).

Similarly, Botman et al. created hiPSCs from 47XXY fibroblasts of Klinefelter syndrome patients and established that the apoptosis markers caspase3 and LDH were present in these cells, causing reduced differentiation efficiency (as evidenced by increased expression of MAGEA and BOLL) (Botman et al. 2020).

Perhaps these techniques can be used to investigate other causes of NOA, such as Kallmann’s syndrome, and other common infertility-causing diseases, such as endometriosis and polycystic ovary syndrome (PCOS). Other promising therapeutic applications for IVG using iPSCs include but are not limited to 1) fertility preservation for prepubertal children needing to undergo gonadotoxic chemotherapy (Pampanini et al. 2021); 2) autologous mitochondrial enrichment using in vitro*-*derived GV oocytes to improve IVF outcomes (Labarta et al. 2019; Easley et al. 2013); and 3) although improbable at present, the generation of gametes for POI and NOA patients, thus removing the need for gamete donation (Cohen et al. 2017). IVG is, however, still experimental, and many of its potential clinical applications are currently still remote (Hendriks et al. 2019).

## Current challenges of IVG using iPSCs

The challenges that face IVG using iPSCs are largely issues of efficiency, safety and ethicality (Saitou and Hayashi 2021; Nishikawa et al. 2008; Stirparo et al. 2018). The “holy grail” of IVG research is to recapitulate the whole human gametogenesis process in vitro using defined factors only and without the need for xenogeneic transplantation (Irie et al. 2015). To date, this has not been achieved, and with current in vitro models, even the PGCLC induction phase has low efficiency (Makar and Sasaki 2020). Moreover, the epigenetic and genomic integrity of in vitro gametes is substantially lower than that of gametes generated in vivo (Irie et al. 2015).

Another limitation that IVG using iPSCs must overcome is safety (Makar and Sasaki 2020). Long-term culture of these cells may elicit epigenetic alterations and methylation profiles (Rao 2008). It has been shown that hiPSCs in prolonged culture have a diminished DNA repair capacity, including a lowered ability to recognize genome damage and decreased coping strategies (Simara et al. 2017). These may result in unknown health issues, including cancer, that can be passed on to future generations (García-Rodríguez et al. 2019). It is therefore imperative to test the quality and integrity of iPSC-derived gametes before they can be put to any clinical use (Saitou and Hayashi 2021).

There are also ethical and social concerns that must be considered when using iPSC-derived in vitro gametes. Although clinical therapeutics using in vitro*-*derived gametes are still remote, with the rate at which science and medicine are progressing, some clinical applications may arrive sooner than expected (Cohen et al. 2017). There are concerns that the widespread application of IVG for reproduction will challenge the most traditional concepts of family, namely, how parenthood is defined and how it is achieved (Cohen et al. 2017; Notini et al. 2020). For example, IVG could open the possibility of generating embryos from two gametes derived from same-sex parents, single individuals, and even deceased individuals (Notini et al. 2020). Other ethical issues include the concern surrounding eugenics and the possibility for “designer babies” (Segers et al. 2019), the commercialization of IVG (Cohen et al. 2017), and germline genome editing using CRISPR technology (Makar and Sasaki 2020).

## Conclusion

From its proof-of-concept studies, the science of IVG has advanced at a very rapid pace. The ground-breaking discovery of iPSCs and their use for the creation of these gametes have contributed enormously to this progress. Several IVG techniques and models using iPSCs have been developed in both mice and humans. Despite this rapid advancement, the efficient and reliable recapitulation of gametogenesis in its entirety in vitro still eludes us. Although oogenesis has been achieved entirely in vitro in mice, the efficiency of the process and the rates of successful term births of the resulting pups remain low. Emerging techniques such as the use of synthetic embryos and the direct induction of germ cells using transcription factors may improve the efficiency of IVG using iPSCs and could facilitate the complete in vitro reconstitution of mature human gametes. Although some scientists believe that the translational applications of IVG are limited to better elucidating germ cell biology, it is already being used to model certain conditions that cause infertility in order to better understand the underlying pathogenetic mechanisms on a cellular level, with the aim of discovering therapeutic options for diseases that are currently untreatable. IVG holds great potential for reproductive medicine and could usher in the next era of reproduction and regeneration. Nevertheless, as with any breakthrough technology, along with this enormous therapeutic potential comes great risk and many ethical responsibilities. The scientific community should continue to cautiously advance technology related to IVG and iPSCs and pay special attention to improving the efficiency and safety of the processes, while also ensuring that they are ethically sound.

## Data Availability

No new data were generated or analysed in support of this review.

## Abbreviations

2i: 2 Kinds of inhibitors
4i: 4 Kinds of inhibitors
ActA: Actin assembly-inducting protein
ALI: Air-liquid interface
AMH: Anti-mullerian hormone
AZFc: Azoospermic factor c
Bex1: Brain expressed x-linked 1
bFGF: Basic fibroblast growth factor
Blimp: B-lymphocyte induced maturation protein 1
BMP: Bone morphogenetic proteine
BOLL: BOULE-like
BOULE: Drosophila boule gene
BPE: Bovine pituitary extract
BVSC: Blimp1-mVenus and Stella-ECFP
c-Myc: Cellular myelocytomatosis oncogene
CRISPR: Clustered regularly interspaced short palindromic repeats
DAZL: Deleted in azoospermia-like germ-cell specific RNA binding protein
DDX4: DEAD-box helicase 4
DNA: Deoxyribonucleic acid
DPPA3: Developmental pluripotency associated 3
Dynll 1: Dynein light chain LC8-type1
EB: Embryoid body
EGF: Epidermal growth factor
EGFP: Enhanced green fluorescent protein
EOMES: Eomesodermin
EpiLCs: Epiblast-like cells
epiPGCs: Epiblast-derive primordial germ cells
EpiSCs: Epiblast stem cells
EPS: Extended pluripotent stem cells
ESCs: Embryonic stem cells
Esrrb: Estrogen-related receptor beta
ETS: Erythrobast transformation specific
Etv5: E-twenty-six (erythrocyte transformation specific) variant gene 6
ETX: Epsilon toxin inducer
FACS: Fluorescence-activated cell sorting
Figla: Factor in germline alpha
FLCs: Follicle-like cells
FOSLCs: Fetal ovarian somatic cell-like cells
FR10Cs5: 10 μM forskolin, 10 μM rolipram, 5 μM cyclosporin A
FSH: Follicle stimulating hormone
GDF9: Growth differentiation factor 9
Gfra1: GDNF (Glial cell line-derived neurotrophic factor) family receptor alpha 1
GSCLCs: Germline stem cell-like cells
GSCs: Germline stem cells
GSK: Glycogen synthase kinase
GSK3: Glycogen synthase kinase 3
GV: Germinal vesicle
H19: 19^Th^ clone in row H imprinted domain
hESCs: Human ESCs
hiPSCs: Human induced pluripotent stem cells
hPGCLCs: Human primordial germ cell-like cells
hPGCs: Human primordial germ cells
HSVtk: Herpes simplex virus thymidine kinase
iBLCs: Blastocyst-like cysts / iBlastoids
ICM: Inner cell mass
ICSI: Intracytoplasmic sperm injection
iMeLCs: Incipient mesoderm-like cells
iPSCs: Induced pluripotent stem cells
IrOvaries: Isogeneic reconstituted ovaries
IVA: In vitro Activation
IVDi: In vitro Differentiation
IVF: In vitro Fertilization
IVG: In vitro Gametogenesis
IVGr: In vitro Growth
IVM: In vitro Maturation
JNK: C-Jun N-terminal kinase
Klf: Krüppel-like factors
KSR: Kinase suppressor of ras
LDH: Lactate dehydrogenase
Lhx8: LIM (*lin-11, Isl-1, mec* 3) homeobox 8
LIF: Leukemia inhibitory factor
Lin28: Cell lineage protein 28
MAGEA: Melanoma-associated antigen family A
MAPK: Mitogen-activated protein kinase
MEK: Mitogen-activated protein kinase kinase
mEpiSCs: Mouse epiblast stem cells
mESCs: Mouse embryonic stem cells
miPSCs: Mouse induced pluripotent stem cells
miR- 294: Micro-RNA 294
miRNAs: Miro ribonucleic acids
MIXL1: Mix paired-like homeobox
mPGCLCs: Mouse primordial germ cell like cells
mPGCs: Mouse primordial germ cells
mPSCs: Mouse pluripotent stem cells
mTSCs: Trophoblast stem cells from mice
mVenus: Membrane-targeted Venus
NOA: Nonobstructive azoospermia
NOBOX: Newborn ovary homeobox
Nr5A1: Nuclear receptor subfamily 5 group A member 1
Oct4: Octamer binding transcription factor 4
OSKM: Oct4, Sox2, Klf4 and c-Myc,
Otx2: Orthodenticle Homeobox 2
Pax1: Paired box 1
PCOS: Polycystic ovary syndrome
PGCLCs: Primordial germ cell like cells
PGCs: Primordial germ cells
PLZF: Promyeolocytic leukemia zinc finger
pMaster12: Plasmid Master 12 episome
POI: Premature ovarian insufficiency
PFT: Primordial to primary follicle transition
Prdm14: Positive regulatory domain containing protein 14
PSCs: Pluripotent stem cells
RFP: Red fluorescent protein
RNA: Ribonucleic acid
rOvaries: Reconstituted ovaries
rOvarioids: Reconstituted ovarioids
rTestes: Reconstituted testes
SC: Stella-controlled ECFP
SCF: Stem cell factor
SCID: Severe combined immunodeficiency
SCP3: Synaptonemal complex protein 3
SLCs: Spermatid-like cells
SMAD: Suppressor of mothers against decapentaplegic
SNRPN: Small nuclear ribonucleoprotein polypeptide N
Sohlh1: Spermatogenesis and oogenesis specific basic helix-loop-helix1
SOX17: Sperm determining region Y-box 17
Sox2: Sperm determining region Y-box 2
SP5: Specificity protein 5
SSC: Spermatogonial stem cells
SSEA1: Stage-specific embryonic antigen-4
Stat3: Signal transducer and activator of transcription 3
STRA8: Stimulated by retinoic acid 8
T1: Primary transitional
Tbp12: TATA box (Goldberg-Hogness box) binding protein 12
Tbx3: T-box transcription factor 3
tdTomato: Tandem dimer Tomato fluorescent protein
Tead2: TEA (*tef-1,abaA*) domain transcription factor 2
TET: Ten-eleven translocation
TF: Transcription factor
Tfap2C: Transcription factor AP-2 Gamma
TSCs: Trophoblast stem cells
Vasa: Vas ATP-dependent RNA helicase
WNT3a: Wingless-related integration site family member 3A
Xq: Long arm of X-chromosome
Zfp42: Zinc finger protein 42
ZP2: Zona pellucida glycoprotein 2
